# Demographic and Social-Cognitive Factors Associated with Weight Loss in Overweight, Pre-diabetic Participants of the PREVIEW Study

**DOI:** 10.1007/s12529-018-9744-x

**Published:** 2018-08-20

**Authors:** Sylvia Hansen, Maija Huttunen-Lenz, Diewertje Sluik, Jennie Brand-Miller, Mathijs Drummen, Mikael Fogelholm, Teodora Handjieva-Darlenska, Ian Macdonald, Alfredo J. Martinez, Thomas Meinert Larsen, Sally Poppitt, Anne Raben, Wolfgang Schlicht

**Affiliations:** 1 Department of Exercise and Health Sciences, University of Stuttgart, Stuttgart, Germany; 2 Division of Human Nutrition, Wageningen University, Wageningen, Netherlands; 3 Charles Perkins Centre and School of Life and Environmental Biosciences, University of Sydney, Sydney, Australia; 4 Department of Human Biology, Maastricht University, Maastricht, Netherlands; 5 Department of Food and Environmental Sciences, University of Helsinki, Helsinki, Finland; 6 Department of Pharmacology and Toxicology, Medical University of Sofia, Sofia, Bulgaria; 7 MRC/ARUK Centre for Musculoskeletal Ageing Research, National Institute for Health Research (NIHR) Nottingham Biomedical Research Centre, Division of Physiology, Pharmacology and Neuroscience, School of Life Sciences, Queen’s Medical Centre, Nottingham, UK; 8 Center for Nutrition Research, University of Navarra, Pamplona, Spain; 9 CIBERobn, Madrid, Spain; 10 IMDEAfood, Madrid, Spain; 11 Department of Nutrition, Exercise and Sports, University of Copenhagen, Copenhagen, Denmark; 12 Human Nutrition Unit, School of Biological Sciences, University of Auckland, Auckland, New Zealand

**Keywords:** Weight loss, Social-cognitive factors, Behavioral determination, Lifestyle intervention

## Abstract

**Purpose:**

Weight loss has been demonstrated to be a successful strategy in diabetes prevention. Although weight loss is greatly influenced by dietary behaviors, social-cognitive factors play an important role in behavioral determination. This study aimed to identify demographic and social-cognitive factors (intention, self-efficacy, outcome expectancies, social support, and motivation with regard to dietary behavior and goal adjustment) associated with weight loss in overweight and obese participants from the PREVIEW study who had pre-diabetes.

**Method:**

Prospective correlational data from 1973 adult participants were analyzed. The participants completed psychological questionnaires that assessed social-cognitive variables with regard to dietary behavior. Stepwise multiple regression analyses were performed to identify baseline demographic and social-cognitive factors associated with weight loss.

**Results:**

Overall, being male, having a higher baseline BMI, having a higher income, perceiving fewer disadvantages of a healthy diet (outcome expectancies), experiencing less discouragement for healthy eating by family and friends (social support), and lower education were independently linked to greater weight loss. When evaluating females and males separately, education was no longer associated with weight loss.

**Conclusion:**

The results indicate that a supportive environment in which family members and friends avoid discouraging healthy eating, with the application of a strategy that uses specific behavior change techniques to emphasize the benefits of outcomes, i.e., the benefits of a healthy diet, may support weight loss efforts. Weight loss programs should therefore always address the social environment of persons who try to lose body weight because family members and friends can be important supporters in reaching a weight loss goal.

## Introduction

Diabetes is a leading cause of mortality and morbidity worldwide. The global age-standardized prevalence of diabetes has nearly doubled since 1980. Globally, an estimated 422 million adults suffered from diabetes in 2014 [1], which is estimated to increase to 642 million by 2040 [2]. Type 2 diabetes mellitus (T2D) accounts for approximately 85 to 95% of all diabetes cases [3]. The prevalence of pre-diabetes [4], which is a precursor to T2D, has also increased globally. Pre-diabetes is characterized by impaired glucose tolerance (IGT) and/or impaired fasting glucose (IFG) and is a condition closely tied to obesity, which is one of the most important risk factors for T2D.

Available evidence has suggested that a promising strategy for the prevention of T2D is adherence to a healthy lifestyle, mainly addressing diet and physical activity [5]. Research has shown that reducing body weight can be effective in preventing and delaying the onset and deterioration of both pre-diabetes and T2D. The US Diabetes Prevention Program (DPP) has indicated that participants with IGT who participated in a lifestyle intervention group reduced their diabetes risk by 58% [6]. In the Look AHEAD (Action for Health in Diabetes) study, a multicenter randomized controlled trial, overweight diabetic participants in the lifestyle intervention group who achieved > 7% weight reduction showed cardio-metabolic improvements, i.e., lower blood pressure, reduced LDL-cholesterol, lower Hb_A1C_ values, and even some remission of diabetes [7].

However, changing a lifestyle is complex and often fails. Lifestyle interventions among adults with diabetes have shown only modest success in maintaining weight loss over the longer term [8]. Generally, people who attempt to lose weight lose less than 5% of their body weight during a period of 12 months or longer [9], and the success of lifestyle interventions in supporting weight maintenance differs between individuals. For example, in the DPP, only 40% of participants achieved the 7% weight loss goal [6]. In the Tuebingen Lifestyle Intervention Program (TULIP), a high-risk phenotype was identified that displayed low insulin secretion relative to insulin resistance or insulin resistant non-alcoholic fatty liver disease, and a low-risk phenotype was an individual without these traits. The TULIP study indicated less improvement in glucose tolerance and a lower reversal rate of pre-diabetes in the high-risk phenotype. This suggests that stratification may help determine the effectiveness of a lifestyle intervention program [10]. Furthermore, a post hoc analysis of the Look AHEAD trial suggests that participants with certain characteristics (e.g., HbA_1c_ or general health) may benefit more from a lifestyle intervention aimed at weight loss than other subgroups [11]. Men are usually underrepresented in weight loss interventions [12] whereas gender differences may exist with regard to the mechanisms that lead to weight loss [13].

Although different health behavior theories have been used to explain weight management, social-cognitive factors associated with weight loss remain poorly understood (e.g., [14, 15]). The relationships between correlates of weight loss differ across study populations, making research in this field more complex. Although many studies have included overweight and obese participants [16], studies that identify correlates of weight loss among high-risk populations, such as pre-diabetic adults, are rare.

Using theories to explain and design behavior change interventions has been increasingly promoted [17, 18]. A number of “social-cognitive theories,” such as the Health Action Process Approach (HAPA) [19], the Social-Cognitive Theory (SCT) [20], the theory of planned behavior (TPB) [21], or the Self-determination Theory (SDT) [22], have chosen and tested a combination of psychological constructs to explain behavior change. In this study, the strength of behavioral intention, self-efficacy, outcome expectancies, self-regulation, and social support as potential factors associated with weight loss is assessed.

In the TPB in particular, intention is considered the most important and proximal predictor of behavior. In turn, intention is influenced by self-efficacy (here defined as the belief to be able to follow the diet even if there are obstacles to overcome) and outcome expectancies (here defined as the expected outcomes with regard to following the diet). Outcomes of behavior can be judged as positive, i.e., expected advantages of certain behaviors (e.g., weight loss and diabetes risk reduction), or negative outcomes, i.e., expected disadvantages of certain behaviors (e.g., being hungry, loss of energy). Particularly during the initial stages of behavior change, outcome expectancies and intention and self-efficacy are important factors in reaching one’s goal. In addition to these factors, social support has been shown to have a strong and positive impact on behavioral change [23]. The exact mechanism through which social support influences behavior is not yet clear. Some studies addressing dietary interventions suggest that social support acts indirectly, e.g., by moderating self-efficacy (cf. [24]).

Once motivated to initiate a specific behavior, the type of self-regulation necessary to stick to that behavior has been indicated as an important mechanism (volitional phase). Self-determination theory differentiates between extrinsic and intrinsic modes of motivation [25] depending on the type of self-determination (non-regulation, external, introjected, identified, integrated, and intrinsic). Being more intrinsically (or autonomously) motivated has been associated with a better adoption of healthy diets [26]. Therefore, only autonomous motivation was included in this study. Further, self-regulation seems to be of particular importance in the initial process of the behavioral change (cf. [27]). When pursuing a goal such as losing body weight, it is important to make an effort to avoid temptations and to stick to behaviors that are helpful in reaching this goal. However, when a previously defined goal becomes unattainable, it becomes a burden on resources [28]. According to Wrosch et al. [34], to give up an unattainable goal and reengage one’s efforts toward a new, but attainable, goal could be an adaptive self-regulatory response [29, 30]. This strategy is called goal adjustment (i.e., giving up on an unattainable goal and re-engaging in an attainable goal). Goal adjustment has been shown to be positively associated with mental health and well-being [31] because it spares cognitive and emotional energy.

Many studies have examined social-cognitive variables from a single theory to explain behavior change. However, studies that focus on a single theory can only explain a small amount of the common variance in weight loss, which is assumed to be multi-determined. To better understand weight loss, it seems beneficial to investigate a combination of psycho-social variables. Such an approach has been used previously by other researchers such as Palmeira et al. [32]. They analyzed variables from four different theories, which explained up to 30% of the common variance of short-term weight loss.

Evidence [16, 33–36] has indicated that self-efficacy, outcome expectancies, self-regulation, and perceived social support and goal adjustment are potential correlates during the initial stages of weight loss, and these factors have therefore been chosen in this sub-study of the PREVIEW intervention trial. There is less evidence for goal adjustment in the framework of weight loss; however, goal adjustment seems to play an important part in goal pursuit. Based on the previous literature [37], participants who experience barriers that make the goal seemingly unattainable will finally not pursue the goal any longer because of decreasing interest and incentives to reach this goal. In PREVIEW, which is an acronym for a large, multicenter, randomized controlled trial (RCT) called “PREVention of diabetes through lifestyle intervention and population studies in Europe and around the world” [38], weight loss is a substantial goal to be reached. Therefore, goal adjustment has been included as a potential correlate of weight loss.

The PREVIEW study aims to reduce the risk of developing type 2 diabetes in a population of overweight and obese adults with pre-diabetes by changing participants’ health behaviors toward eating a healthy diet and being physically active on a regular basis. As part of the PREVIEW trial, an evidence-based and theory-oriented group-counseling program called *PRE*VIEW behavior *M*odification *I*ntervention *T*oolbox (PREMIT) [39] was designed to support the participants’ behavior change. PREMIT has several stages based on the Transtheoretical Model (TTM) [40] to guide participants from behavioral initiation to maintenance. PREMIT incorporates variables of different health behavior theories (e.g., [41]). The social-cognitive variables examined in this study were targeted within the PREMIT program to support participants in their behavior change efforts.

This paper analyzes data from the low-energy-diet (LED) phase of the PREVIEW RCT. Weight loss is an intervening variable for the primary endpoint (incidence of T2D) of PREVIEW, so the analysis here addressed a secondary endpoint, i.e., weight loss, a proximal determinant of pre-diabetes and T2D.

The current study aimed to identify demographic and social-cognitive correlate variables in men and women for weight changes that could potentially be targeted by behavioral interventions aiming to support body weight loss. It is hypothesized that higher intention, stronger self-efficacy, more expectations of benefits, and lower expected disadvantages of a healthy diet, in addition to higher motivation and social support from family and friends at the start of the weight-reduction phase of the PREVIEW trial, predict higher weight loss eight weeks later. Furthermore, analyses using goal adjustment as a potential correlate of weight loss were also performed. It is hypothesized that better goal adjustment (being able to disengage from unattainable goals and reengage in new goals) is associated with greater weight loss. It is expected that the ability to disengage from an unattainable goal and reengage in a new goal is associated with greater weight loss.

## Methods

### Intervention Procedure

PREVIEW is registered in clinicaltrials.gov [NCT01777893] and was conducted at eight study sites: the University of Copenhagen (Denmark), the University of Helsinki (Finland), the University of Maastricht (The Netherlands), the University of Nottingham (UK), the University of Navarra (Spain), the Medical University of Sofia (Bulgaria), the University of Sydney (Australia), and the University of Auckland (New Zealand). PREVIEW consisted of two phases: (1) an 8-week weight reduction phase using a commercial (Cambridge weight plan) LED (3400 kJ/daily) to achieve ≥ 8% weight loss, followed by (2) a weight loss maintenance phase (34 months), in which participants were randomized into four different intervention arms. During the weight reduction phase, the participants took part in PREMIT group sessions. In these sessions, they learned behavior change strategies and received advice on how to achieve the ≥ 8% weight loss goal, which was defined as the criterion for eligibility to the weight maintenance phase of the PREVIEW trial. On the repeated clinical investigation days (CIDs), the participants’ anthropometrics, hemodynamic (e.g., blood pressure), and metabolic (e.g., Hb_A1c_) values were registered. The participants also answered a battery of questionnaires and scales on social-cognitive variables using an online platform.

### Participant Recruitment

A total of 2326 eligible participants were recruited at the 8 study centers between June 2013 and February 2015. Participants eligible for the study were overweight (BMI > 25 kg/m^2^) and pre-diabetic adults between 25 and 70 years old. Pre-diabetes was diagnosed by an oral glucose tolerance test (OGTT) according to the American Diabetes Association criteria (ADA): (i) IFG, with a venous plasma glucose concentration of 5.6 to 6.9 mmol/l when fasting, and/or (ii) IGT, with a venous plasma glucose concentration of 7.8 to 11.0 mmol/l at 2 h after the oral administration of a standard 75 g glucose dose, and fasting plasma glucose < 7.0 mmol/l. Individuals diagnosed with T2D were excluded. Detailed inclusion and exclusion criteria and a detailed description of the study protocol have been published elsewhere [38].

## Measurements and Procedures

This study used demographic, social-cognitive, and anthropometric data from all participants measured on CID 1 (the start of the weight reduction phase). Body weight loss in kilograms was calculated as the difference in body weight between CID 1 and CID 2. Sociodemographic variables including the participants’ sex, age, educational status (low educational status: no education–secondary vocational education, high educational status: higher vocational education and university education), and total household income (low income: less than 39.100€ and high income: 39.100€ and above) were collected through a self-administered questionnaire on CID 1. The income categories were adjusted for each country to account for country differences.

Social-cognitive variables were measured using standardized questionnaires. All questionnaires were prepared in English, translated into the local language (i.e., Finnish, Danish, Dutch, Spanish, and Bulgarian), and then back-translated to English by authorized translators. Any translational difficulties were discussed and resolved.

### Intention

The participants were asked to assess their intentions to “eat as healthy as possible.” The item was adapted from Renner and Schwarzer [42] and rated using a scale from 1 (I don’t intend at all) to 7 (I strongly intend). Inherent to the recruitment procedure, all participants showed strong intentions. In the statistical analysis, intention was entered as a dichotomized variable: very high intention (7) vs. anything less than very high intention (1 to 6), because even through data transformation, the normality of the variable could not be improved.

### Self-efficacy

The participants were asked how convinced they were that they could stick to a healthy diet (I can manage to stick to a healthy diet) using a questionnaire with five items each (e.g., even when I have worries and problems; even if I need a long time to develop the necessary routines) [43]. The participants rated their persuasiveness of self-efficacy on a scale ranging from 1 (very uncertain) to 4 (very certain). The mean value was computed, with low scores reflecting low self-efficacy and high scores reflecting strong beliefs. The self-efficacy scale showed an excellent internal consistency (Cronbach’s *α* = 0.91).

### Goal Adjustment

The participants used the self-regulation of goal adjustment scale [44]. The goal disengagement dimension of the scale asked the participants about the ease with which they were able to reduce efforts (two items) and relinquish commitment toward unattainable goals (two items) (e.g., If I have to stop pursuing important goals in my life … it is easy for me to reduce my effort toward the goal). To measure goal reengagement (e.g., If I have to stop pursuing important goals in my life … I start working on other new goals), the participants rated a second dimension of the scale, the extent to which they generally reengage in new goals if they face constraints on goal pursuits (six items). The response options ranged from 1 (strongly disagree) to 5 (strongly agree). For both goal disengagement and goal reengagement, the mean scores were computed. Cronbach’s *α* for the goal re-engagement scale and the goal disengagement scale were *α* = 0.88 and 0.71, respectively. A factor analysis confirmed both dimensions. The Kaiser-Meyer-Olkin measure verified the sampling adequacy (KMO = 0.85), and Bartlett’s test of sphericity was significant. An acceptable level of explained variance of 61.5% was reached.

### Outcome Expectancies

The outcome expectancy of behavior change was assessed for healthy eating and included 12 items (e.g., I eat healthy foods; I will not have weight problems anymore) using subscales from Renner and Schwarzer [42]. The participants rated expected benefits and disadvantages with regard to their behavioral change from 1 (not at all true) to 4 (exactly true). The mean values for benefits and disadvantages were computed. Low scores reflect fewer expected benefits/disadvantages, and high scores reflect more expected benefits/disadvantages. The internal consistencies for outcome expectancies-benefit of a healthy diet and for disadvantages of a healthy diet were Cronbach’s *α* = 0.70 and *α* = 0.67, respectively. Both dimensions were confirmed. The Kaiser-Meyer-Olkin measure verified the sampling adequacy (KMO = 0.72), and Bartlett’s test of sphericity was significant. An acceptable level of explained variance of 51% was reached.

### Self-regulation of Motivation

The Treatment Self-Regulation Questionnaire (TSRQ) [45] was used to ask the participants about their motivation to eat healthy. The questionnaire includes 15 items and covers reasons why participants (would) follow a healthy diet. The scale discriminates between different self-regulatory modes: autonomous (six items; e.g., Why do you follow a healthy diet? Because a healthy diet is consistent with my life goal), introjected (two items; e.g., Why do you follow a healthy diet … because I would feel bad about myself if I ate unhealthy food), externally (four items, e.g., … because I want others to approve of me) and a-motivation (three items, e.g., … I do not really know why I should eat healthy). The responses were given on scales ranging from 1 (not at all true) to 7 (very true). In the statistical analysis, only autonomous motivation was used and was entered as a dichotomized variable: very high motivation (7) vs. anything less than very high motivation (1 to 6) as the autonomous mode displayed the strongest association with weight (e.g., [26]). Autonomous motivation for dietary behavior showed excellent reliability with a Cronbach’s *α* of 0.92. The dimension as classified by the authors of the original instruments was confirmed. The Kaiser-Meyer-Olkin measure verified the sampling adequacy (KMO = 0.85), and Bartlett’s test of sphericity was significant. An acceptable level of explained variance of 71.5% was reached. For this study, only the subscale for autonomous motivation was used.

### Received Social Support for Dieting

Perceptions of received self-reported social support for dietary behavior were assessed by asking the participants about their support from family and friends using the Social Support for Diet and Exercise Behavior Scale [46]*.* The scale by Sallis, Grossman, Pinski, Patterson, and Nader [46] has been used by several studies to measure “perceived” social support [47, 48]. However, the scale measures the actual received support [49, 50]. Received social support measures “[… ]are thought to more accurately reflect actual support provided by the environment than other types of social support” [51].

The participants rated ten statements about their families’ and friends’ encouragement and discouragement regarding the participant’s dietary behavior (e.g., During the past two months, my family or friends (assessed separately) encouraged me not to eat unhealthy foods when I am tempted to do so). The items were rated on a scale from 1 (none) to 5 (very often). In case the question did not apply to the participant, an additional response option was given (does not apply).

A factor analysis using the PREVIEW data verified the sampling adequacy (KMO = 0.80), and Bartlett’s test of sphericity was significant. The dimensions “encouragement for diet by family” and “encouragement for diet by friends” as classified by the authors of the original scale were confirmed. The analysis did not differentiate between discouragement from family and discouragement from friends as suggested by the authors of the original scale. The items “ate unhealthy food in front of me” (*λ* = 0.783 (family); *λ* = 0.763 (friends)), “brought home foods I am trying not to eat,” and “offered me food I am trying not to eat” (*λ* = 0.710 (family); *λ* = 0.630 (friends)) loaded on one factor, disregarding whether family or friends were addressed. The items “refused to eat the same foods I eat” (*λ* = 0.666 (family); *λ* = 0.661 (friends)) and “got angry when I encouraged them to eat healthily” (*λ* = 0.785 (family); *λ* = 0.774 (friends)) loaded on another factor, also independent of whether family or friends were addressed. Following the dimensional analyses based on the PREVIEW data, the items were grouped into four dimensions: (1) family encouragement for diet, (2) friends encouragement for diet, (3) temptations to eat unhealthily by family and friends, and (4) discouragement to eat healthily by family and friends. An acceptable level of explained variance of 65.5% was reached. Cronbach’s *α* for social support—encouragement by family/friends reached *α* = 0.88 and *α* = 0.77, respectively, and Cronbach’s α for temptations by family and friends and discouragement to eat healthy by family and friends reached *α* = 0.85 and *α* = 0.80, respectively.

### Weight Loss

Body weight in kilograms (kg) was measured by a study nurse on CID 1 and CID 2. The participants were lightly clad without wearing shoes. The criterion variable weight loss was used as a continuous variable and calculated as the total weight loss (in kg) between CID 1 and CID 2. The mean weight loss was 10.70 kg (± 3.77).

## Statistical Methods

Prior to the statistical analyses, extreme outliers (> mean ± 3 SD) were removed from the dataset. Missing data for any of the social-cognitive variables were imputed using the multiple imputation method with the fully conditional specification model (Markov chain Monte Carlo). Ten duplicate datasets were generated.

Descriptive statistics were used to describe the socioeconomic characteristics of the participants, reported as the mean ± SD. Differences between the participants who finished and those who did not finish the LED phase (dropouts) were analyzed by an independent-sample *t* test/chi-square test with Bonferroni’s correction (*p* < 0.005).

Correlations between social-cognitive variables collected on CID 1 and weight loss were assessed by Pearson’s correlation coefficient. A stepwise multivariate linear regression was executed with variables significantly correlated to examine the association with weight loss. The analysis was performed using data from the total sample and for women and men separately.

The following variables were included: age, sex (only used in the analysis for the total sample), BMI at baseline, education, income, goal reengagement, goal disengagement, outcome expectancies (benefits of a healthy diet, disadvantages of a healthy diet), motivation (autonomous motivation for a healthy diet), and social support (encouragement for changing eating habits by family and encouragement for changing eating habits by friends, temptations to eat unhealthy by family and friends, discouragement to eat healthy). All variables were measured on CID 1.

Sensitivity analyses were performed to assess the robustness of the results; these results were only reported if they differed between the original and imputed data. The statistical analyses were conducted with IBM® SPSS Statistics Program version 24.

## Results

Of the 2326 eligible participants, 2224 individuals began the LED phase. Weight data on CID 2 were available for 2020 participants. Forty-seven cases were excluded from the analyses because of extreme outliers (> mean ± 3 SD). Therefore, data from 1973 participants were analyzed.

The means and standard deviations measured on CID 1 are shown in Table 1. The participants ranged from 25 to 70 years old with a mean age of 52.14 years (± 11.35). In total, 1324 of the participants were female and 649 were male. The mean BMI was 34.95 kg/m^2^ (± 5.94). Higher education (i.e., higher vocational education or higher) was reported by 1080 participants (55%). Additionally, 794 participants had a household income (before tax) lower than 39.100 €, whereas 1049 participants had a household income greater than 39.100 €. Data from dropouts (*n* = 157) differed significantly (*p* < 0.01) from those of the finishers. The dropouts were younger (*M* = 45.77 years, SD = 12.02; *t* = − 7.23), had a higher baseline BMI (*M* = 37.22 kg/m^2^, SD = 7.79; *t* = 3.26), a lower income (low income 48%, high income 37.7%; *χ*^2^ = 9.84), higher scores for outcome expectancies (*M*_disadvantage of a healthy diet_ = 2.13, SD = 0.57; *t* = 3.09), and higher scores for social support (*M*_encouragement from family_ = 3.12, SD = 1.17; *t* = 3.25); *M*_encouragement from friends_ 2.49, SD = 1.12; *t* = 3.75; *M*_discouragement to eat healthy by family and friends_: 1.99, SD = 0.98; *t* = 3.36).

**Table 1:** Baseline characteristics of the study participants

Demographic and social-cognitive variables	Percentage (%) or mean ± SD
Age (in years) (*N* = 1973)	52.14 ± 11.35
Sex (%)	
Men	32.9
Women	67.1
Education (*n* = 1751)^a^	
Low (secondary vocational education or lower) (%))	33.8
High (higher vocational education or higher) (%))	55.0
Income (*n* = 1852)^a^	
Low (≤ 39,100 €) (%))	41.0
High (> 39,100 €) (%))	52.9
BMI at baseline (weight in kg/(height in m^2^)) (*N* = 1973)	35.10 ± 6.34
Intention for a healthy diet (*N* = 1973)	
Low (*n* (%))	27.9
High (*n* (%))	72.1
Self-efficacy for healthy diet (range 1–4) (*N* = 1973)	3.13 ± 0.57
Goal disengagement (range 1–5) (*N* = 1973)	2.83 ± 0.77
Goal reengagement (range 1–5) (*N* = 1973)	3.49 ± 0.73
Outcome expectancies (*N* = 1973)	
Benefits of a healthy diet (range 1–4)	3.34 ± 0.46
Disadvantages of a healthy diet (range 1–4)	1.99 ± 0.53
Autonomous motivation for a healthy diet (range 1–7) (*N* = 1973)	
Low (*n* (%))	41.3
High (*n* (%))	58.7
Social support (*N* = 1973)	
Encouragement for changing eating habits by family (range 1–5)	2.84 ± 1.16
Encouragement for changing eating habits by friends (range 1–5)	2.20 ± 0.97
Temptations to eat unhealthy by family and friends (range 1–5)	2.59 ± 0.89
Discouragement to eat healthily by family and friends (range 1–5)	1.80 ± 0.80

^a^For income and education data were available for N participants (*N* = 1852 and *N* = 1751, respectively) due to missing data

### Correlations

The Pearson correlation coefficients between variables and weight loss are summarized in Table 2. Significant (*p* < 0.05) positive correlations were found between goal disengagement and weight loss and between encouragement for changing eating habits by family and weight loss. Furthermore, the disadvantages of a healthy diet and discouragement to eat healthily by family and friends were negatively correlated with weight loss (*p* < 0.05). All correlations between social-cognitive variables were low.

**Table 2:** Correlation matrix of social-cognitive variables and weight loss (kg)

	Weight loss	1	2	3	4	5	6	7	8	9	10	11	12
1. Gender	0.38**												
2. BMI at baseline	0.35**	− 0.06**											
3. Intention for a healthy diet	− 0.00	− 0.14**	0.09**										
4. Self-efficacy for healthy diet	0.02	− 0.03	0.03	0.24**									
5. Goal disengagement	0.04	− 0.02	0.02	− 0.05*	− 0.10**								
6. Goal reengagement scale	− 0.04	− 0.03	− 0.07**	0.04	0.11**	0.26**							
7. Autonomous motivation for healthy diet	− 0.04	− 0.13**	0.00	0.26**	0.27**	− 0.02	0.08**						
8. Benefits of a healthy diet	0.01	− 0.09**	0.06**	0.20**	0.26**	− 0.06**	0.07**	0.34**					
9. Disadvantages of a healthy diet	− 0.08**	0.02	0.11**	− 0.05*	− 0.13**	− 0.02	− 0.01	− 0.08**	0.07**				
10. Encouragement for changing eating habits by family	− 0.06**	0.09**	0.12**	− 0.00	0.09**	− 0.03	0.02	0.06*	0.18**	0.10**			
11. Encouragement for changing eating habits by friends	− 0.04	− 0.05*	0.19**	0.05*	0.08**	− 0.03	0.04	0.05*	0.17**	0.12**	0.577**		
12. Temptations to eat unhealthy by family/friends	− 0.05*	− 0.11**	0.10**	0.04	− 0.02	− 0.02	0.00	0.01	0.06**	0.20**	0.199**	0.220**	
13. Discouragement to eat healthily by family/friends	− 0.10**	− 0.06**	0.11**	0.04	0.03	− 0.02	− 0.02	− 0.04	0.02	0.23**	0.244**	0.335**	0.481**

*p < 0.05; **p < 0.01

### Stepwise Linear Regression Analysis

Table 3 shows the best predictive model for weight loss of the total sample after 8 weeks (phase 1, LED period), which explains 32% of the common variance. The model indicated that male sex (*β* = 0.38), BMI (*β* = 0.41), income (*β* = 0.11), expectation of disadvantages of a healthy diet (*β* = − 0.10), discouragement to eat healthily by family and friends (*β* = − 0.10), and education (*β* = − 0.05) were the most important and significant correlates of weight loss (*p* < 0.05).

**Table 3:** Predictive model explaining the weight change (in kg) after LED period

Correlate	*B*	SE *B*	*β*	*R*^2^ change	*p*
Constant	3.25	0.53			
Sex (men)	3.04	0.16	0.38	0.14	< 0.001
Baseline BMI	0.24	0.01	0.41	0.14	< 0.001
Disadvantages of a healthy diet	− 0.70	0.15	− 0.10	0.02	< 0.001
Income (high)	0.81	0.16	0.11	0.01	< 0.001
Discouragement to eat healthily by family and friends	− 0.45	0.10	− 0.10	0.01	< 0.001
Education (high)	− 0.36	0.16	− 0.05	0.00	< 0.05
Final adjusted *R*^2^	0.32				

Overall *F* = 131.31, *p* < 0.001

The best predictive model for weight loss for women and men separately is shown in Table 4; this model explains 23 and 18% of the common variance, respectively. The model for women indicated that BMI (*β* = 0.47), expectation of disadvantages of a healthy diet (*β* = − 0.11), discouragement to eat healthily by family and friends (*β* = − 0.11), and income (*β* = 0.11) were the most important and significant correlates of weight loss (*p* < 0.05).

**Table 4:** Predictive model explaining the weight change (in kg) after LED period separately for women and men

Correlate	*B*	SE *B*	*β*	*R*^2^ change	*p*
Women					
Constant	3.20	0.54			
Baseline BMI	0.23	0.01	0.47	0.20	< 0.001
Disadvantages of a healthy diet	− 0.61	0.15	− 0.11	0.02	< 0.001
Discouragement to eat healthily by family and friends	− 0.40	0.10	− 0.11	0.01	< 0.001
Income (high)	0.67	0.16	0.11	0.01	< 0.001
Final adjusted *R*^2^				0.23	
Overall *F* = 87.95, *p* < 0.001					
Men					
Constant	50.10	10.07			
Baseline BMI	0.28	0.02	0.42	0.15	< 0.001
Disadvantages of a healthy diet	− 0.72	0.32	− 0.09	0.02	< 0.001
Discouragement to eat healthily by family and friends	0.91	0.34	− 0.12	0.01	< 0.005
Income (high)	− 0.65	0.22	0.10	0.01	< 0.005
Final adjusted *R*^2^				0.19	
Overall *F* = 33.15, *p* < 0.001					

The model for men indicated that BMI (*β* = 0.42), expectation of disadvantages of a healthy diet (*β* = − 0.09), discouragement to eat healthily by family and friends (*β* = − 0.12), and income (*β* = 0.10) were the most important and significant correlates of weight loss.

## Discussion

The primary aim of this paper was to identify correlates of weight loss in a sample of pre-diabetic, overweight participants enrolled in the PREVIEW RCT. The multiple regression model for the total sample showed that being male, having a higher income, having a lower educational status (i.e., secondary vocational education or lower), and having a higher baseline BMI were associated with greater weight loss during an 8-week fixed-intake LED program. A combination of modifiable social-cognitive variables was also associated with greater weight loss, specifically fewer expected disadvantages from eating a healthy diet and less discouragement to eat healthily by family and friends. Separate analyses for women/men showed very similar results. Education was significant in the total sample; however, it did not reach the level of significance in the model for women or in the model for men.

Previous studies have consistently found that baseline BMI and sex influence weight loss [33]. Considering that the LED phase of PREVIEW is a diet with a fixed energy intake, it is expected that individuals with a higher baseline body weight and male participants will lose more body weight. Thus far, the results here are not surprising. However, the association between income and overweight/obesity found here has not yet been established [52].

A study by Madji and colleagues [53] indicated that obesity and diabetes risk are independent of educational status. Contrary to previous studies investigating the relationship between sociodemographic factors and BMI (e.g., [54, 55]), in our study, a lower educational status predicted higher weight loss when assessing the entire sample. However, this association disappeared when the women’s and men’s data were analyzed separately. One explanation could be that more educated individuals may have more sedentary jobs than less educated participants. Sedentariness leads to less energy expenditure during most of the day, hindering weight loss.

Although most studies have not found an association between outcome expectancies and weight loss [16, 32, 56], a study by Carels and colleagues [57] found positive associations between intra-individual perceived benefits (outcome expectancies) and weight loss. In a study by Anderson, Winett, and Wojcik [58], disadvantages, called negative outcome expectancies by the authors, were associated with higher fat, but lower fiber, fruit, and vegetable intakes. Our results suggest, as expected, that participants who expected fewer disadvantages of a healthy diet lost more weight than those who expected more disadvantages of a healthy diet. Expecting fewer disadvantages may be more important to the PREVIEW participants in the process of weight loss than expecting benefits. Therefore, avoidance motivation might be a stronger predictor than approach motivation [59].

Dietary and physical activity interventions mobilizing social support have resulted in more weight loss than interventions without a social support component [34]. In addition, “enhancing social support” has been found to have a strong relationship with BMI reduction in people with recently diagnosed diabetes [60]. Other studies have found social support to be an important correlate, particularly for weight maintenance; however, it is less important as a correlate for successful weight loss [33]. The significant result of received social support in the current study emphasizes the importance of family and friends in weight loss because the discouragement of healthy eating by family and friends was negatively associated with weight loss.

Several studies have found self-efficacy and autonomous motivation to be positively associated with weight loss [32, 56, 61]. Unexpectedly, we did not observe this result in our study. A possible explanation for not finding a significant association between self-efficacy and weight loss in our study is that a change in dietary self-efficacy during weight loss may better predict weight loss than baseline self-efficacy scores (cf. [14]) or that self-efficacy may better predict long-term weight control [62, 63]. Another explanation is given by Teixeira and colleagues [61]. They suggested that generalized measures of self-efficacy might be better correlates of weight loss than eating-related self-efficacy.

That autonomous motivation was not significantly associated with weight loss may be because healthy eating is not inherently pleasurable, particularly in the context of a phase in which energy intake is limited. Eating patterns may rather be directed toward external outcomes such as losing weight or attaining a more desirable physique or improving health [64]. Thus, in the early stages of behavior change, more extrinsic forms of motivation may be more predictive of weight loss.

### Strengths and Limitations

On a methodological note, since social-cognitive factors were assessed at baseline and related to weight loss during the LED phase, causality cannot be established. Furthermore, little variance and ceiling effects in data gathering within the participant population sample may possibly explain the insignificant results for intention, for which most of the participants had reported high values. This was likely due to a participant selection bias. Ceiling effects may vanish over time, and the data from future CIDs may better predict the effect of the PREVIEW intervention on weight in PREVIEW participants. In addition, intention was only measured using a single item.

The strengths of this study are the external validity due to the study’s naturalistic setting and the large number of overweight pre-diabetic participants. However, there are methodological limitations. Cronbach’s alphas indicated that not all scales had a satisfactory reliability. Self-efficacy, goal adjustment, motivation, and social support showed acceptable to good reliabilities. However, the reliabilities for outcome expectancies were lower. This might have been due to the heterogeneous participant sample that included participants from, e.g., different countries. Reliabilities could not be improved when single items were deleted, which is a common method to enhance the reliability of a scale. Low reliability may lead to an underestimation of correlations. In addition, the participants were very eager to start the intervention, as concluded from their autonomous motivation and intention values. Both variables were dichotomized because a normal distribution could not be achieved by transforming the values. However, dichotomizing variables results in reduced statistical power. Most of the participants were women who had obtained a high level of education and income. This does not allow generalizing the results to populations with different socioeconomic backgrounds. Additionally, self-reported data are vulnerable to bias. Body weight and loss of body weight are influenced by energy intake and energy expenditure. Assumptions can be made about the social-cognitive variables and their associations with weight loss during the weight loss phase, but not with dietary behaviors. In addition, no data were collected indicating different volumes of physical activity and sedentary time. Both could have moderated or even mediated the weight loss. In our study, the participants were not given any specific instructions on the volume of physical activity during the LED phase.

## Conclusion

Because of the study design, it is not possible to draw a causal conclusion. However, this study provides some valuable information. In addition to previously identified variables associated with weight loss (i.e., sex and higher initial body weight), social support, and perceived benefits of outcome expectancies facilitate weight loss in overweight, pre-diabetic persons. The fact that all associations were weak was presumably a result of the complexity of behaviors causing weight loss. Behaviors leading to weight loss are influenced by a combination of psychological, social, and environmental variables that are mutually interrelated and dynamic over time.

It can be assumed, based on the data, that weight loss interventions targeting overweight adults with pre-diabetes could benefit from behavior change techniques that aim to change outcome expectancies and to ensure that the benefits of a healthy diet can be made clear. Furthermore, the social environment (family and friends) should be integrated into the intervening strategy. Strengthening a supportive environment seems very important to reach the goal. Nevertheless, this concept remains speculation. We will address these presumptions during the PREVIEW intervention study that assesses effects on weight maintenance.
